# Adverse Events Following COVISHIELD Vaccination Among Adult Population in Bangladesh

**DOI:** 10.1007/s42399-021-01021-z

**Published:** 2021-07-31

**Authors:** Md. Musab Khalil, Khandker Mahbub-Uz-Zaman, As-Saba Hossain, Farid Ahmed, Md. Fazlul Karim Chowdhury, Sharmin Tahmina Khan, Md. Shah Alam Miah, Narwana Khaleque, Md. Golam Kibria, Faruque Ahmed, Ahad Mahmud Khan

**Affiliations:** 1 Sheikh Russel Gastroliver Institute and Hospital, Dhaka, Bangladesh; 2 American International University of Bangladesh, Dhaka, Bangladesh; 3 The University of Edinburgh, Edinburgh, UK

**Keywords:** COVID 19, Vaccination, Adverse Events, COVISHIELD, Oxford AstraZeneca

## Abstract

The study aimed to determine how frequently the adverse events of the COVISHIELD vaccine occur among the Bangladeshi population. This cross-sectional study was conducted at Sheikh Russel Gastroliver Institute and Hospital, Mohakhali, Dhaka, Bangladesh, in May 2021. The inclusion criteria were the adult populations who received the 2nd dose of the COVISHELD vaccine and had passed 28 days following the completion of the 2nd dose. Three hundred and five persons fulfilling the inclusion criteria were asked over the telephone—based on a predesigned questionnaire. The rates of adverse events were 54.1% and 41.3% after the 1st and 2nd dose of vaccine, respectively, and the difference was statistically significant (*p* < 0.001). Pain at the injection site was the most common adverse event (32.5% following the 1st dose and 27.9% following the 2nd dose). All of the symptoms were mild and lasted for about 2 days. Age and comorbidities were significantly associated with the adverse events (*p* < 0.001). Neither doses had any vaccine-related life-threatening adverse event nor had any symptoms related to vaccine-related blood clotting. Nineteen persons (6.2%) had been diagnosed with COVID-19 after the 1st dose of vaccination, and three (1%) persons had been diagnosed with COVID-19 after the 2nd dose of vaccination. As no significant life-threatening adverse event was observed, this study might help reduce the hesitancy for vaccination among the population and thus help reduce transmission of this highly contagious virus.

## Introduction

Coronavirus disease 2019 (COVID-19) has significantly damaged the world population with mortality and morbidity of unprecedented scale. As of 03 May 2021, more than 150 million people have been confirmed to be identified as being infected with the disease, and about 3.2 million people succumbed to death because of this disease [1]. COVID-19 is caused by SARS-COV-2, an enveloped, positive-sense single-stranded RNA virus, which has a glycoprotein structure resembling spikes on the surface, responsible for the attachment with receptor and cellular invasion of the host [2, 3]. Patients diagnosed with COVID-19 usually suffer from fever, cough, and shortness of breath, along with other symptoms. Although predominantly a respiratory tract infection, it affects other organs as well. The death of the patients was attributed to the development of multiple organ failure. Dysregulated immune-thrombosis involving neutrophils and platelets has been designated as the reason for increased incidents of acute respiratory distress syndrome (ARDS) and systemic hypercoagulability [4–6].

Currently, there is no specific treatment for this deadly disease. Implementation of different prevention modalities has been put in place to interrupt the transmission of the disease. Vaccination is a key feature among these modalities. As of 04 May 2021, 13 different vaccines are being used globally. Of these, two are mRNA vaccines, four are vector (non-replicating) vaccines, five are inactivated, and two are protein subunit vaccines. Over one billion dosages of vaccines have been administered [7]. It has been shown that frontline workers are at risk of contracting the infection because of the limited supply of personal protective equipment (PPE) [8]. Hence, for the safeguard of the healthcare workers and to ensure the interruption of the transmission cycle, vaccination holds the best approach for the prevention and control of the disease [9].

Bangladesh introduced vaccination for the vulnerable groups, frontline workers, and emergency personnel on 27 January 2021 and nationwide vaccination drive from 07 February 2021 with the vaccine COVISHEID based on ChAdOx1 nCoV-19 vaccine that has been developed by The Jenner Institute, Oxford University, with the technical support from AstraZeneca and being produced by Serum Institute of India, Pune, Maharashtra, India. Bangladesh purchased this vaccine from the Serum Institute of India [10].

This ChAdOx1 nCoV-19 vaccine consists of the replication-deficient simian adenovirus vector ChAdOx1, containing the full-length structural surface glycoprotein (spike protein) of SARS-CoV-2, with a tissue plasminogen activator leader sequence. ChAdOx1 nCoV-19 expresses a codon-optimized coding sequence for the spike protein [11]. Bangladesh is to receive a total number of 68 million doses of vaccine as a target of vaccinating the 30% vulnerable population as well as protecting the frontline workers [12]. As this is an unprecedented event, it is important to follow up the safety of the vaccine for all the adverse events to be recorded and stored, and also keep track of the severe adverse events following the vaccination as part of vaccine surveillance so that any damage could be minimized and adequate steps could be taken beforehand.

Adult vaccination drive has never been undertaken on such a scale. Therefore, the acceptability of the vaccine as well as the safety concern regarding the vaccine has to be evaluated. Even though there has been some acceptability, vaccine hesitancy, defined as the vaccine acceptance is prolonged or refusal to accept the service of vaccination, is a feature among the people [13]. In a cross-sectional study, it has been shown to have 61.34% acceptability of the vaccine, but only 35.14% are willing to accept it [14].

There is also a need to rule out certain misconceptions regarding the side effects of the vaccine that need to be addressed to prevent vaccine hesitancy. Therefore, this study was conducted among vaccinated persons regarding the post-vaccination adverse events and severe adverse events for the safety of the vaccinated persons as well as for future policy planning and implementation.

## Methods

### Study Design, Setting, and Participants

This cross-sectional study was conducted at Sheikh Russel Gastroliver Institute and Hospital (SRGIH), Mohakhali, Dhaka, Bangladesh, from 8 to 15 May 2021. A total of 350 persons aged 18 years and older received the 2nd dose of *COVISHIELD vaccine*, and in 28 days passed from the last dose, they were approached over the telephone.

### Sampling Technique

A list of persons who completed the 2nd dose of vaccine and completed 28 days post-vaccination was obtained from the hospital records. A consecutive sampling technique was applied. These 350 vaccine recipients were approached over the phone to collect data from them. At least three attempts of calling were made at least 1 h apart before telling non-response. Forty-five persons did not respond to the call. Finally, 305 persons were enrolled.

### Data Collection

Data collection was done between the 30th and 35th day following 2nd dose of vaccination. The participants were asked over the telephone using a predesigned structured questionnaire. Data regarding sociodemographic characteristics, comorbidities, adverse events of vaccination (both the doses), and diagnosis of COVID-19 were collected. The types of adverse events collected were local, general, medically attended, and serious adverse events. The local adverse events included pain, swelling, itching, and rash at the injection site. General adverse events included fever, chills and rigors, generalized body ache, fatigue, malaise, somnolence, drowsiness, insomnia, headache nausea, vomiting, diarrhea, dizziness, joint pain, runny nose, and redness of the eyes. Medically attended adverse event was defined if a medical practitioner was consulted for managing the adverse events. An event was considered serious if it resulted in death, was life-threatening, required in-patient hospitalization or prolongation of existing hospitalization, resulted in persistent or significant disability/incapacity, or was a congenital anomaly/birth defect [15]. The severity of the adverse events was assessed by numerical rating scale (NRS) starting from 0 to 10 [16]. Zero was depicted as having no symptom, 1–3 as mild symptoms, 4–6 as having moderated symptoms, and 7–10 as severe symptoms [17]. The diagnosis of COVID-19 was based on RT PCR for SARS-COV-2 test [18].

### Statistical Analysis

Collected data were cleaned and assessed for distribution. All quantitative data were non-normally distributed except the age of the study participants. Quantitative data were expressed in mean ± SD, and qualitative data were expressed in percentage. Wilcoxon paired signed ranks test was used to compare paired data. Mann-Whitney *U* test was used to compare two groups of quantitative variables. Binary logistic regression was done to assess the predictor of adverse events of vaccination. A two-tailed value of *p* ≤ 0.05 was considered statistically significant for all analyses.

## Results

Among the 305 participants, 173 were males (56.4%), with their mean age being 47 years (SD 13.6) (Table 1). The rates of adverse events were 54.1% and 41.3% after the 1st and 2nd doses of vaccine, respectively. The adverse events were significantly higher following the 1st dose compared to the 2nd dose (*p* < 0.001). The most frequent adverse event was pain at the injection site that was reported among 99 (32.5%) after the 1st dose and 85 (27.9%) after the 2nd dose. Fever was recorded among 61 (20%) in 1st dose and 37 (12.1%) in the 2nd dose. Fatigue, malaise, generalized body ache, and headache were recorded 6.9%,16.4%, 16.4%, and 6.9%, respectively, following the 1st dose of vaccination, while after the 2nd dose, these were reported 3.3%, 8.2%,9.5%, and 5.2% which showed the adverse events occurred in significantly less during the second vaccination (*p* < 0.001). However, none of the participants had severe life-threatening adverse events. None of them had symptoms related to thrombosis as well. The symptoms were mild based on the NRS that lasted mostly 2 days. Furthermore, almost one-fifth of the participants needed medications, namely paracetamol, to get rid of the symptoms. Few participants had to consult with the medical practitioners (8.2% after the 1st dose and 3.9% following the second dose). Significant numbers of the participants had their daily activities hampered following the 1st dose in contrast to the second dose (*p* < 0.007 vs. *p* < 0.832). Additionally, the severity and duration of adverse events and adverse events hampering daily activities were more with the 1st dose, and the difference was statistically significant (Table 2).

**Table 1:** Sociodemographic factors and comorbidities (*n* = 305)

Age, mean ± SD (in years)		47.3 ± 13.6
Gender, *n* (%)	Male	173 (56.7)
	Female	132 (43.3)
Occupation, *n* (%)	Doctor	35 (11.5)
	Nurse	19 (6.2)
	Pharmacist	6 (1.9)
	Healthcare professional (other than doctor, nurse, and pharmacist)	19 (6.2)
	Service	80 (26.2)
	Business	43 (14.1)
	Housewife	75 (24.6)
	Retired from service	11 (3.6)
	Others*	17 (5.6)
Smoking habit, *n* (%)	Smoker	40 (13.1)
	Ex-smoker	21 (6.9)
	Non-smoker	244 (80)
Comorbidity, *n* (%)	Present	152 (49.8)
	Absent	153 (50.2)

*Engineer, chartered accountant, architect, school teacher, banker, driver

**Table 2:** Adverse events after vaccination (*n* = 305)

		After 1st dose		After 2nd dose		p*
Adverse events occurred, *n* (%)		165 (54.1)		126 (41.3)		< 0.001
Pain at injection site, *n* (%)		99 (32.5)		85 (27.9)		
Fever, *n* (%)		61 (20)		37 (12.1)		
Fatigue, *n* (%)		21 (6.9)		10 (3.3)		
Malaise, *n* (%)		50 (16.4)		25 (8.2)		
Generalized body ache, *n* (%)		50 (16.4)		29 (9.5)		
Headache, *n* (%)		21 (6.9)		16 (5.2)		
Chills and rigor, *n* (%)		9 (3)		5 (1.6)		
Swelling at injection site, *n* (%)		9 (3)		2 (0.7)		
Itching and rash at injection site, *n* (%)		0 (0.0)		1 (0.3)		
Dizziness, *n* (%)		3 (1)		4 (1.3)		
Nausea, n (%)		8 (2.6)		5 (1.6)		
Vomiting, *n* (%)		2 (0.7)		2 (0.7)		
Diarrhea, *n* (%)		2 (0.7)		1 (0.3)		
Somnolence, *n* (%)		2 (0.7)		1 (0.3)		
Drowsiness, *n* (%)		1 (0.3)		0 (0.0)		
Runny nose, *n* (%)		2 (0.7)		0 (0.0)		
Redness of the eye, *n* (%)		1 (0.3)		0 (0.0)		
Joint pain, *n* (%)		3 (1)		1 (0.3)		
Insomnia, *n* (%)		0 (0.0)		1 (0.3)		
Severity of adverse event in NRS, mean ± SD		2.6 ± 1.5		2.5 ± 1.6		0.027
Duration of adverse event, mean ± SD		1.9 ± 1.3		1.7 ± 0.9		0.01
Medication needed to manage adverse event, *n* (%)		79 (25.9)		60 (19.7)		0.003
Consulted medical practitioner for managing adverse event, *n* (%)		25 (8.2)		12 (3.9)		0.009
Adverse event hampered daily activity, *n* (%)		70 (23%)		31 (10.2)		< 0.001
Adverse event hampered daily for how many days, mean ± SD		1.6 ± 1.2		1.5 ± 1.2		0.083
		Severity of adverse event in NRS		Severity of adverse event in NRS		
		Mean ± SD	p**	Mean ± SD	p**	
Adverse event hampered daily activity	Yes	3.1 ± 1.8 (*n* = 60)	0.007	2.7 ± 1.9 (*n* = 31)	0.832	
	No	2.3 ± 1.2 (*n* = 105)		2.5 ± 1.4 (*n* = 95)		

*Wilcoxon paired signed ranks test, **Mann-Whitney *U* test, *p* ≤ 0.05 considered significant. *NRS*, numerical rating scale

Moreover, age and comorbidity had been significantly associated with the vaccine-related adverse events (Table 3). Almost half of the participants had comorbidities. Among the comorbid populations, hypertension was the most prevalent (35.4%), followed by diabetes mellitus, which constituted one-fourth of the comorbid population (23.3%). More than two-thirds of them were non-smokers. Following the adverse events, the mean duration of the symptoms persisted 1.9 ± 1.3 days (*p* < 0.01) for the 1st dose of vaccination while 1.7 ± 0.9 days (*p* < 0.01) following the 2nd dose of vaccination.

**Table 3:** Factors predicting adverse events of vaccine (*n* = 305)

	1st dose of vaccine, model *x*^2^ (16.8, *p* 0.005)					2nd dose of vaccine, model *x*^2^ (32.8, *p* < 0.001)			
Factor	Variable coding for factor	*B*	S.E.	OR	_P_μ	*B*	S.E.	OR	_P_μ
Age		0.036	0.011	1.037	0.001	0.060	0.012	1.062	< 0.001
Gender	Male = 1 Female = 2	− 0.432	0.266	0.649	0.104	0.089	0.278	1.093	0.748
Non-smoker		Constant			0.278				0.806
Ex-smoker		− 0.479	0.382	0.619	0.210	− 0.075	0.393	0.928	0.849
Smoker		− 0.603	0.496	0.547	0.225	− 0.338	0.517	0.713	0.514
Comorbidity	Present = 1 Absent =2	0.594	0.278	1.811	0.033	1.226	0.303	3.408	< 0.001

*μ*, binary logistic regression; *p* ≤ 0.05, considered significant; *B*, regression coefficient; *OR*, odds ratio; S.E.-Standard error

**Fig. 1:**
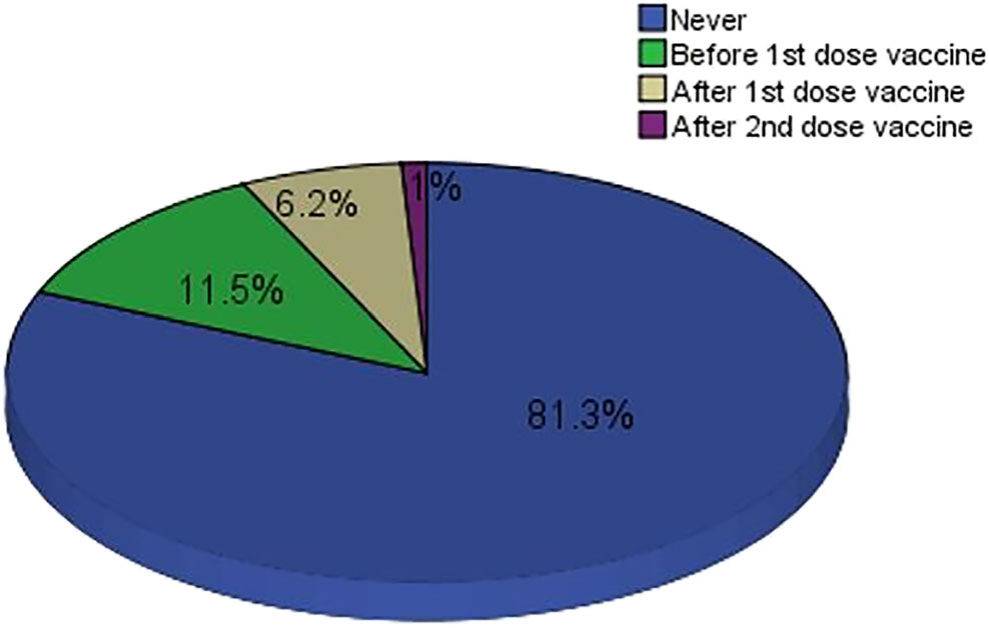
Time of diagnosis of COVID-19 in relation to vaccination, *n* = 305

Nineteen (6.2%) had COVID-19 after the 1st dose, and 3 (1%) participants had COVID-19 after the 2nd dose (Figure 1). Among the participants diagnosed with COVID-19 following vaccination, the majority had been diagnosed with COVID-19 after the second week. None of the patients who suffered from COVID-19 became critically ill (Table 4).

**Table 4:** Diagnosis of COVID-19 after the 1st or 2nd dose of the vaccine (*n* = 22)

		After 1st dose of vaccine (*n* = 19)	After 2nd dose of vaccine (*n* = 3)
Vaccination to symptom onset of COVID-19, *n* (%)	Asymptomatic	1 (5.3)	0 (0.0)
	Within 2 weeks of the vaccine dose	3 (15.8)	1 (33.3)
	After 2 weeks of the vaccine dose	15 (78.9)	2 (66.7)
Duration of symptoms of COVID-19, *n* (%)	No symptom	2 (10.5)	1 (33.3)
	≤ 7 days	13 (68.4)	1 (33.3)
	8–14 days	4 (1.3)	0 (0.0)
	> 14 days	0 (0.0)	1 (33.3)
Treated at, *n* (%)	Home	15 (4.9)	2 (66.7)
	Hospital	4 (21.1)	1 (33.3)
Needed oxygen, *n* (%)	Yes	3 (15.8)	1 (33.3)
	No	16 (84.2)	2 (66.7)
Needed HDU/ICU, *n* (%)	Yes	0 (0.0)	0 (0.0)
	No	19 (100)	3 (100)
Needed intubation, *n* (%)	Yes	0 (0.0)	0 (0.0)
	No	19 (100)	3 (100)

## Discussion

This study demonstrates that the rates of adverse events of the COVISHIELD vaccine were 54.1% after the 1st dose and 41.3% after the 2nd dose of the vaccine. However, these were minor adverse events like pain at the injection site, fever, feeling unwell, and generalized body ache. There were no significant life-threatening adverse events after vaccine administration.

**Figure 2:**
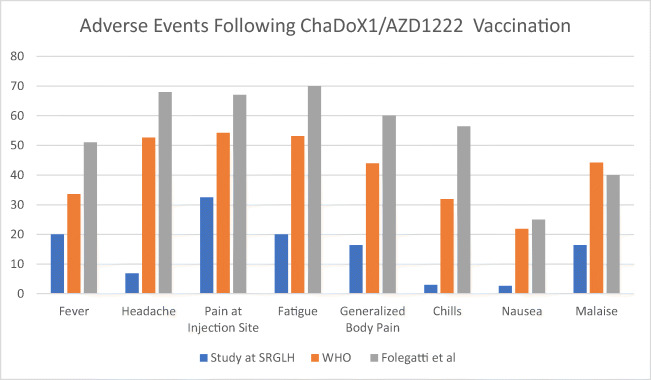
Comparison of adverse events reported in the study with WHO SAGE Working Group AZD-1222 vaccine against COVID-19 and Folegatti et al. [22]

Given the outcome of the phase 3 trial in the United Kingdom (UK), Brazil, and South Africa, the ChadOx1 vaccine had demonstrated 63% efficacy against symptomatic COVID-19 cases, with acceptable tolerability and no serious adverse event related to vaccination [19, 20]. Adverse events recorded in our study were compared to the anticipated adverse events by the World Health Organization (WHO) SAGE working group based on phase 3 clinical trials in the UK, Brazil, and South Africa and phase 1/2 trial in the UK, and there were significant differences [19–21]. In the WHO Working Group, the headache had been observed among 52.6% of participants, while the phase 1/2 trial found 68% of participants [19, 20]. Compared to that, our study found only 6.9% of the study population complained of headache following the vaccination. Another adverse event chill was present among 31.9% by the WHO Working Group paper and 56.4% among the phase 1/2 trial, while our study population only reported 3% following vaccination. Pain at the injection site was present among 32.5% of participants from our study, while compared that with the WHO Working SAGE Working Group paper and phase 1/2 trial in the UK reported 54.2% and 67%, respectively. Nausea as an adverse event following the vaccination had been reported only among 2.6% of our participants; compared that with the WHO SAGE Working Group and phase 1/2 trial in the UK, it was reported 21.9% and 25%, respectively [19–21]. Phase 1/2 trial in the UK found generalized body pain among 60% of participants, and the WHO SAGE Working Group paper anticipated this adverse event to be reported among 44% of the population. In our study, we only recorded 16.4% population reported this adverse event [19–22]. Figure 2 shows the comparison of adverse events reported in our study with the study of the WHO SAGE Working Group [19–21] and Folegatti et al. [22].

When comparing the adverse event from the 1st dose and 2nd dose of vaccination, according to the WHO SAGE Working Group, adverse events that occurred more than 10% are being denoted as very common, and from 1 to 10% are being termed as common. By that classification, pain at the injection site (32.5%), fever (20%), malaise (16.4%), and generalized body ache (16.4%) were the very common adverse events following the 1st dose of vaccination, while fatigue (6.9%), headache (6.9%), chill (3%), swelling at the injection site (3%), dizziness (1%), and nausea (2.6%) were common adverse events in our study. Following the 2nd dose of vaccination, only pain at the injection site (27.9%) and fever (12.1%) are denoted as very common adverse events, and the rest can be described as common adverse events [21, 20].

Our study data following binary logistic regression suggested comorbidity had a significant relation with the adverse event following the 1st and 2nd dose of vaccination with odds ratio for the 1st dose being 1.8 with S.E. 0.278, *p* < 0.005, and for the second dose, odds ratio is 3.408, S.E. 0.303, and *p* < 0.001. Our finding for the duration symptoms persisted more than 7 days for only 4 participants following the 1st dose of vaccination while only 2 participants reported symptoms more than 7 days following the 2nd dose vaccination, which was lower than what the WHO SAGE Working Group showed with the persistence of 13% following day 7 [19]. After the 1st dose of vaccination, the mean duration of adverse events following vaccination is 1.9 ± 1.3 days, while after the 2nd dose of vaccination, the mean duration is 1.6 ± .09 days. The adverse events severity as well persistence had been less in our study population compared to the WHO SAGE Working Group paper based on multiple trials conducted in the UK, South Africa, and Brazil [19, 22, 20].

For the adverse events, only 25 participants consulted the physicians for their symptoms where 79 persons reported to have taken medication for the relief of symptoms and 70 participants reported hampered daily activity, with mean of 1.6 ± 1.3 days following the first dose, compared to 1.5 ± 1.2 days following the 2nd dose. After the 2nd dose, the report of hampering of daily activity had been reported by 31 participants. Like the adverse events which were reported significantly less than the 1st dose, the severity and hampering daily activity showed the same trend. The severity of the adverse event hampering daily activity following 1st dose vaccination had been shown statistically significant mean of 3.1 ± 1.8 days (*p* < 0.007), which was not the case following the 2nd dose. It also solidifies the fact that the severity of the adverse events was significantly less than in the 1st dose of vaccination [20].

### Limitation of the Study

No immediate allergic reaction following vaccination was recorded, and there had not been any incidents of anaphylactic reaction or shock following the vaccination. This also denotes the allergic reaction to the components of the vaccine is minimum, but the scale of the population may give us limited knowledge. During the initial phase of the vaccine development, it has been privy to transverse myelitis and pyrexia, which has not been demonstrated among our study population. Immunothrombosis following vaccination had been reported as a very rare adverse event following vaccination among the population with low platelet count; however, none of the members of the study population had shown any sign of such condition. As these are very rare adverse events, our study population data may be limited in this regard and may require further study with a larger population.

## Conclusion

These observations exemplify the fact that the COVISHIELD vaccine is a well-tolerated and safe vaccine, which can be administered among the adult population of Bangladesh. As no significant life-threatening adverse event was observed, this study might help reduce hesitancy for vaccination among the population and thus help reduce transmission of this highly contagious disease.

## Data Availability

These are available upon reasonable request.
